# Brain network analysis reveals convergent and divergent aberrations between mild stroke patients with cortical and subcortical infarcts during cognitive task performing

**DOI:** 10.3389/fnagi.2023.1193292

**Published:** 2023-07-06

**Authors:** Mengru Xu, Linze Qian, Sujie Wang, Huaying Cai, Yi Sun, Nitish Thakor, Xuchen Qi, Yu Sun

**Affiliations:** ^1^Key Laboratory for Biomedical Engineering of Ministry of Education of China, Department of Biomedical Engineering, Zhejiang University, Hangzhou, China; ^2^Department of Neurology, Sir Run Run Shaw Hospital, Zhejiang University School of Medicine, Hangzhou, China; ^3^Department of Biomedical Engineering, National University of Singapore, Singapore, Singapore; ^4^Department of Neurosurgery, Sir Run Run Shaw Hospital, Zhejiang University School of Medicine, Hangzhou, China; ^5^Department of Neurosurgery, Shaoxing People's Hospital, Shaoxing, China; ^6^State Key Laboratory of Brain-Machine Intelligence, Zhejiang University, Hangzhou, China

**Keywords:** graph theory, functional connectivity, cognitive impairment, mild stroke, EEG

## Abstract

Although consistent evidence has revealed that cognitive impairment is a common sequela in patients with mild stroke, few studies have focused on it, nor the impact of lesion location on cognitive function. Evidence on the neural mechanisms underlying the effects of mild stroke and lesion location on cognitive function is limited. This prompted us to conduct a comprehensive and quantitative study of functional brain network properties in mild stroke patients with different lesion locations. Specifically, an empirical approach was introduced in the present work to explore the impact of mild stroke-induced cognitive alterations on functional brain network reorganization during cognitive tasks (i.e., visual and auditory oddball). Electroencephalogram functional connectivity was estimated from three groups (i.e., 40 patients with cortical infarctions, 48 patients with subcortical infarctions, and 50 healthy controls). Using graph theoretical analysis, we quantitatively investigated the topological reorganization of functional brain networks at both global and nodal levels. Results showed that both patient groups had significantly worse behavioral performance on both tasks, with significantly longer reaction times and reduced response accuracy. Furthermore, decreased global and local efficiency were found in both patient groups, indicating a mild stroke-related disruption in information processing efficiency that is independent of lesion location. Regarding the nodal level, both divergent and convergent node strength distribution patterns were revealed between both patient groups, implying that mild stroke with different lesion locations would lead to complex regional alterations during visual and auditory information processing, while certain robust cognitive processes were independent of lesion location. These findings provide some of the first quantitative insights into the complex neural mechanisms of mild stroke-induced cognitive impairment and extend our understanding of underlying alterations in cognition-related brain networks induced by different lesion locations, which may help to promote post-stroke management and rehabilitation.

## 1. Introduction

Stroke is a widespread, serious, and disabling global healthcare problem (Warlow et al., 2011). In China, stroke is the leading cause of death and one of the primary factors of acquired disability in adults (Wu et al., 2019). In 2017, there are 11.1 million stroke patients in China, with an annual growth of 2.4 million new stroke cases (Wang et al., 2017). Hence, stroke has inevitably imposed a huge disease burden on the Chinese healthcare system, as most patients with stroke will survive the initial illness (Wang et al., 2020b). There are increasing number of research on stroke that aims to investigate the underlying neural mechanisms and seek better methods of treatment and rehabilitation. However, a sizeable amount of research has focused on moderate-to-severe stroke patients with apparent sensorimotor or cognitive deficits (Umarova, 2017; Gassert and Dietz, 2018), while patients with mild stroke have often been neglected due to being assumed can recover with little or no intervention. In particular, previous studies have shown that mild stroke may not be as benign as thought (Smith et al., 2005; Edwards et al., 2006a). A retrospective analysis suggested that physicians may decide not to administer intravenous tissue plasminogen activator to stroke patients with milder symptoms because they believe symptoms are too mild to require treatment, but a considerable number of those patients were unable to be discharged home due to neurological disabilities (Smith et al., 2005). Given that mild stroke accounts for a significant proportion of post-stroke patients (Saa et al., 2019), it is necessary to investigate the characteristics of patients with mild stroke for better rehabilitation.

Cognitive impairment after stroke is prevalent (Rost et al., 2022). In fact, a few studies of cognitive dysfunction in patients with mild stroke have shown that cognitive impairment is also present and can affect an individual's ability to perform activities of daily living (Wolf et al., 2009; Wolf and Rognstad, 2013). For instance, previous research investigated the consequences of patients with mild stroke, focusing on several common cognitive functions (e.g., memory and attention), and found declines in cognitive function and health-related quality of life (Carlsson et al., 2003). In a prospective cohort study designed to identify factors associated with cognitive impairment after stroke, Jacquin et al. (2014) found a high frequency of cognitive impairment in patients with mild stroke. In fact, the type of cognitive impairment is associated with the lesion locations. Lesions of the basal ganglia can impair the performance of all cognitive functions (Kazim et al., 2021), but in clinical practice, cortical lesions are generally considered more severe than subcortical lesions. However, scientific evidence for the impact of cortical and subcortical lesions on cognitive impairment is limited and inconsistent. A study of neuropsychological examination of patients with acute stroke demonstrated an increased risk of common cognitive impairment in patients with cortical strokes, but subcortical involvement was not excluded (Nys et al., 2007). Another study in acute stroke showed that the subcortical patients outperformed the cortical patients in terms of visuospatial/constructional function (Wilde, 2010). Nonetheless, when research focused on cognitive function in subacute stroke patients, no differences were found between patients with cortical and subcortical lesions (Planton et al., 2012). However, none of these studies involved patients with mild stroke. Considering the potential influence of post-stroke cognitive impairment on rehabilitation and functional recovery, as well as the effects of lesion location on stroke rehabilitation (Frenkel-Toledo et al., 2021; Xuefang et al., 2021), extending the study from mild stroke to patients with different lesion locations may help to understand location-related cognitive deficits in mild stroke patients and facilitate subsequent rehabilitation.

In clinical practice, post-stroke cognitive impairment is usually assessed with neuropsychological testing. However, the testing typically involves a battery of neuropsychological tests to provide a comprehensive assessment, which is often complex, time-consuming, and can be biased by different physician instructions (Dejanović et al., 2015). Consequently, there are numerous studies employing short neuropsychological screening tests [e.g., Mini-Mental State Examination (MMSE)] to assess cognitive function (Ballard et al., 2003; Gottesman and Hillis, 2010), which are generally limited in their sensitivity to detect cognitive impairment in stroke patients with a ceiling effect (Barnay et al., 2014). Additionally, MMSE is less sensitive to mild cognitive impairment in stroke patients (Mitchell, 2009), and is no better than chance in detecting cognitive deficits (Bour et al., 2010). Therefore, neuropsychological testing may not be an appropriate measurement tool for mild stroke, nor is it suitable for the impact of different lesion locations on cognitive function in patients with mild stroke. Furthermore, due to their inherent limitations, neuropsychological tools cannot provide information on the underlying mechanisms of cognitive impairment induced by mild strokes. To make a comprehensive understanding of mild stroke-induced cognitive dysfunction and the influence of lesion location on cognitive function, it is not only necessary to evaluate the cognitive impairment of patients with mild stroke, but more importantly, to understand the essential characteristics and mechanisms of their impact on cognitive impairment.

Electroencephalography (EEG) has attracted increasing attention in the measurement of brain activity for its advantages of high temporal resolution, low cost, and non-invasive (Cao et al., 2022). For example, by using resting-state EEG-based power spectral density analysis, asymmetry in spectral power between hemispheres was found in chronic stroke, most pronounced at lower frequencies (Saes et al., 2019). Heuristically, our brain is a highly efficient network, consisting of a large number of different brain regions, each with its own task and function, but constantly exchanging information with each other. In this way, these brain regions form a complex integrative network where information is continuously transmitted and processed between brain regions that are structurally and functionally related: the brain network (Friston et al., 1994; Bullmore and Sporns, 2009; Bassett and Sporns, 2017). Recently, studies of the human brain in terms of connectivity patterns have revealed important information about the structural, functional, and causal organization of the brain. Functional connectivity (FC), which indicates the temporal correlations between neurophysiological events that are spatially distant from each other (Friston et al., 1993), has received increasing attention in the study of neurological diseases in recent years (Liu et al., 2016; Vecchio et al., 2019). Previous brain network studies on stroke revealed that even a well-localized stroke can cause complex clinical symptoms due to the highly interconnected organization of the cerebral cortex (Greicius, 2008; Dørum et al., 2020). Vecchio et al. (2023) recorded EEG signals from patients with acute stroke to explore whether acute stroke would cause alterations in brain network architecture, and their findings demonstrated that global FC changes regardless of whether the hemisphere is affected. Notably, graph theoretical analysis can characterize the FC of complex brain networks by different properties, which has been employed in neuroscience to unveil the stroke-induced changes in functional brain networks from local to global levels (Fallani et al., 2013; Philips et al., 2017). For instance, in a study of patients with chronic stroke, Philips et al. (2017) found that decreased global and local efficiency in the unaffected hemisphere was associated with improved movement after stroke. However, compared with studies of motor recovery, few cognitive-related FC studies to date have been conducted with stroke survivors (Dacosta-Aguayo et al., 2014, 2015). A pilot study of patients with right hemispheric stroke showed that poor cognitive recovery was related to increased resting-state functional activity in the contralateral hemisphere, whereas patients with good cognitive recovery did not differ significantly from healthy controls. The results suggest a strong correlation between changes in FC and cognitive recovery in stroke patients (Dacosta-Aguayo et al., 2014). Previous scattered studies of cognitive decline after stroke have primarily utilized resting-state functional activity (Dacosta-Aguayo et al., 2014, 2015). However, there is still limited evidence on alterations in functional brain networks during cognitive processing and the impact of lesion location on cognitive function in patients with mild stroke.

Taking all the above into consideration, the present study aims to extend cognition-related research to patients with mild stroke and further explore whether there are differences and commonalities in the brain network reorganization induced by lesion location during cognition. Specifically, we adopted graph theoretical analysis to investigate the effect of mild stroke on cognitive function. A widely-used cognition-related paradigm (i.e., the oddball task) with two modalities including visual and auditory information was employed in this work. Furthermore, to investigate the effect of lesion location on cognitive function, we applied statistical analysis based on graph theory measurements from global to node scales to explore the relationship between lesion location and brain network properties during cognitive processes. Since previous studies have repeatedly mentioned that cognitive impairment is common in stroke patients (Wolf and Rognstad, 2013; Rost et al., 2022), we, therefore, hypothesized that mild stroke would lead to cognitive impairment, which could be reflected in behavioral performance and network analysis. Moreover, the evidence of cognitive impairment caused by different lesion sites is inconsistent (Wilde, 2010; Planton et al., 2012). We further hypothesized that distinct patterns of brain network reorganization could be observed between cortical and subcortical patient groups. To this end, we recorded the EEG signals of two mild stroke patient groups (i.e., patients with cortical stroke and patients with sub cortical stroke) and age-matched healthy controls during two cognitive tasks (i.e., visual and auditory oddball tasks), EEG functional connectivity was estimated using the weighted phase lag index (wPLI) of each pair of electrodes in four widely used frequency bands. Subsequently, the intrinsic properties of functional brain networks were quantitatively assessed by graph theoretical analysis to explore the neural mechanisms related to cognition and whether patients with cortical and subcortical stroke would exhibit divergent and convergent patterns of topological reorganization of the functional brain network.

## 2. Materials and methods

### 2.1. Participants

A group of 88 outpatients with mild stroke was enrolled during routine annual call-back health checkup at the Department of Neurology, Sir Run Run Shaw Hospital, Zhejiang University School of Medicine. Inclusion criteria were: (1) age > 18 years; (2) clinically diagnosed as mild stroke symptoms [National Institute of Health Stroke Scale (NIHSS) (Brott et al., 1989): <5]; (3) no obvious visual and hearing deficiency; (4) without any concomitant neurological or psychiatric problems; (5) able to follow commands and accomplish tasks independently. The included patients were divided into two groups [Cortical Stroke (CS)/Subcortical Stroke (SS) = 40 / 48] according to the lesion locations determined by experienced physicians through examining the computed tomography (CT)/magnetic resonance imaging (MRI). Specifically, patients in the CS group present lesions located at the frontal, parietal, temporal, and occipital areas; whereas, the SS group had lesions in the internal capsule, thalamus, basal ganglia, and cerebellum. In addition, 50 age and gender-matched healthy subjects recruited from the local community were included as normal controls (NC). Demographics of healthy controls and stroke patients were shown in Table 1. The Institutional Review Boards of the Sir Run Run Shaw Hospital, Zhejiang University School of Medicine approved the protocol, and each participant provided written informed consent in accordance with the Declaration of Helsinki.

**Table 1:** Demographics of normal controls and stroke patients.

	**NC (*n* = 50)**	**CS (*n* = 40)**	**SS (*n* = 48)**	***p*-value**
Gender (M/F)	19/31	22/18	24/24	0.243^a^
Age (years)	63.26 ± 6.21	64.93 ± 10.91	62.19 ± 10.33	0.385^b^
Education (years)	6.62 ± 3.98	7.50 ± 4.35	6.85 ± 4.17	0.566^c^
Handedness (R/L)	48/2	40/0	48/0	0.168^a^
Time after stroke (months)	–	2.40 ± 5.80	1.84 ± 7.55	0.117^d^

Values for age, education, and time after stroke are presented as mean ± SD.

Values for gender and handedness are presented as ratios.

a Chi-Square test was used for independence between two categorical variables.

b One-way ANOVA test was used to test for differences in the means of the three groups.

c Kruskal-Wallis test was used as a nonparametric test for comparing three independent samples.

d Mann-Whitney *U*-test was used to compare the differences between two independent samples when the sample distributions are not normally distributed.

### 2.2. Experimental paradigms

**Figure 1:**
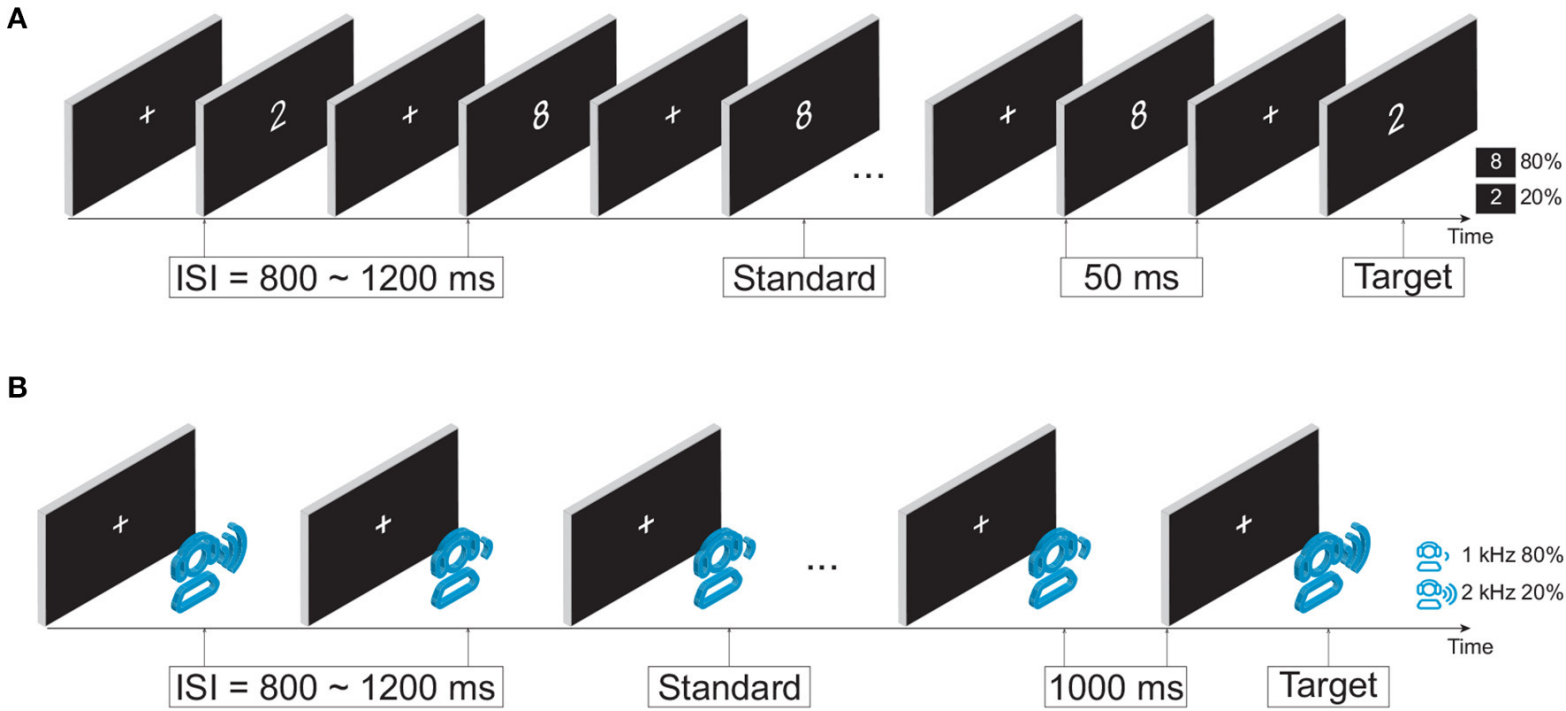
Experimental design. **(A)** Visual oddball paradigm. **(B)** Auditory oddball paradigm.

Taking into account the potential cognitive impairment in mild stroke patients, this study aimed to investigate the intrinsic task-dependent functional brain network changes. Two previously validated attention tasks (i.e., visual and auditory oddball tasks) were used to provide an appropriate and participant-friendly cognitive paradigm (Figure 1). During both tasks, each participant sat in front of a computer monitor at a distance of 60 cm in a quiet room. Participants were instructed to respond to target stimuli on the button response pad with the index finger of their dominant hand. Specifically, press the left-most button on the response pad during the visual task, and press the second button from the left during the auditory task.

#### 2.2.1. Visual oddball task

In the visual oddball paradigm, as illustrated in Figure 1A, the infrequent target and frequent standard stimuli were a white number 2 (113 × 175 pixels) and a white number 8 (113 × 171 pixels) in the center of the monitor, respectively. The occurrence probabilities of target and standard stimuli were 20% (100 stimuli in total) and 80% (400 stimuli in total). The duration of the stimulus was fixed at 50 ms with a random interstimulus interval (ISI) of 800−1,200 ms. The total duration of the visual task was ~9 min.

#### 2.2.2. Auditory oddball task

In the auditory oddball paradigm, as shown in Figure 1B, an infrequent target tone at 2,000 Hz and a frequent standard tone at 1,000 Hz were randomly presented. The occurrence probabilities of the target tone and standard tone were 20% (40 stimuli in total) and 80% (160 stimuli in total), respectively. The duration of stimuli was fixed at 1,000 ms with a random ISI of 800–1,200 ms. The auditory stimuli were delivered via a headset device (Philips SHP2000). The total duration of the auditory task was around 7 min.

### 2.3. EEG recordings and data pre-processing

EEG signals were recorded by using a 60-channel NeuroScan SynAmps2 amplifier (Model: 8050, Compumedics Neuroscan USA) according to the international 10–20 system and digitized with a sampling rate of 1,000 Hz. The reference electrode was located between CPZ and CZ (the location of the reference electrode in the Quid-Cap supplied with the SynAmps2). Vertical and horizontal electrooculogram (EOG) data were recorded from additional electrodes, which were placed above/below the left eye to measure the vertical ocular artifacts, and lateral to the external canthi to measure the horizontal ocular artifacts. Electrode impedances were kept below 5 kΩ during data collection and a 50 Hz notch filter was employed to avoid the main interferences. A previously-validated EEG preprocessing pipeline was adopted (Gao et al., 2022), including down-sampling to 256 and 1–40 Hz bandpass filtering. Notably, any choice of reference point will inevitably affect EEG measurements, since the human body does not have a neutral point (Nunez and Srinivasan, 2006). To eliminate this effect, the data are subsequently re-referenced to the average potential over the entire head, the average reference, which approximates the voltages relative to infinity (Nunez et al., 2001). The average reference has the advantages of a simple computational process and low cost, and is generally considered to be a fairly neutral reference whose performance is not altered due to the addition of noise (Chella et al., 2016). Then bad epochs were removed by visual inspection. Independent component analysis (ICA) was employed to eliminate artifacts from muscle activities, and ocular artifacts by removing the highly correlated components with EOG signals (Jung et al., 2000). After that, EEG signals were segmented into [−100, 500] ms (0 ms denotes the beginning of the target stimulus), where [−100, 0] ms data was used for baseline correction. Off-line analysis were carried out using in-house scripts and EEGLAB toolbox (Delorme and Makeig, 2004) in MATLAB 2021a (The MathWorks Inc., USA).

### 2.4. Network connectivity

To estimate phase synchronization between signals, weighted phase lag index (wPLI) was employed to evaluate FC and construct brain networks. As an extension of the phase lag index (PLI) (Stam et al., 2007), wPLI has been proven to be less sensitive to uncorrelated noise (Sunwoo et al., 2017). By weighing each phase difference according to the magnitude of the lag, phase differences close to zero contribute little to the calculation of the wPLI. Thus, in the case of volume-conducted noise sources with near-zero phase lag, wPLI increases the sensitivity to capture true changes in phase synchronization (Vinck et al., 2011). Specifically, the instantaneous phase of the signal was obtained by Hilbert transform. Let *x*_*j*_(*t*) denotes the time series of the *j*-th channel, the analytical signal *X*_*j*_(*t*) is calculated as:


(1) $$\begin{array}{l} X_{j}\left(t\right)=x_{j}\left(t\right)+i{\bar{x}}_{j}\left(t\right), \end{array}$$

where ${\bar{x}}_{j}\left(t\right)$ is the Hilbert transform of *x*_*j*_(*t*). Let *X*_*k*_(*t*) indicates the analytical signal of the *k*-th channel, and the complex cross-spectrum between the two channels can be expressed as:


(2) $$\begin{array}{l} Z\left(t\right)=X_{j}\left(t\right)X_{k}{\left(t\right)}^{*}, \end{array}$$

where $X_{k}{\left(t\right)}^{*}$ denotes the complex conjugate of *X*_*k*_(*t*). wPLI is calculated based on the imaginary component of the cross-spectrum:


(3) $$\begin{array}{l} wPLI=\frac{\left|E\left\{ℑ\left(Z\right)\right\}\right|}{E\left\{ℑ\left(Z\right)\right\}}=\frac{\left|E\left\{|ℑ\left(Z\right)|sgn\left(ℑ\left(Z\right)\right)\right\}\right|}{E\left\{\left|ℑ\left(Z\right)\right|\right\}}, \end{array}$$

where |·| denotes the absolute value, ℑ(*Z*) refers to the imaginary component of *Z*, and *sgn* denotes sign function. wPLI values range from 0 to 1, e.g., a value of 0 indicates no phase synchronization, while a value of 1 means full synchronization between two channels.

To obtain specific frequency properties of brain networks, EEG signals were band-pass filtered into delta (1–4 Hz), theta (4–7 Hz), alpha (8–12 Hz), and beta (13–30 Hz) frequency bands. wPLI was then computed for all electrode pairs in each frequency band for all [0, 500] ms epochs. Therefore, for each participant, task, frequency band, and epoch, a 60 × 60 weighted adjacency matrix was constructed. Subsequently, the adjacency matrices were averaged across epochs to obtain the final adjacency matrix for each participant. Thus, there were four 60 × 60 adjacency matrices per task per participant.

### 2.5. Graph theoretical analysis

According to the graph theoretical approach, the brain can be represented as a graph constructed from a set of nodes (e.g., EEG electrodes) and edges (e.g., functional connections), which indicate that there are interactions between pairs of nodes (Stam and Reijneveld, 2007). During complex cognitive tasks, the brain needs to regulate the flow of information by making a trade-off between integrating and segregation of incoming stimuli (Deco et al., 2015). In the concept of graph theoretical analysis, the integration and segregation of information in the brain are manifested in the network topology as the tendency to be organized in clusters, and the ability to exchange information between different regions, respectively (Cohen and D'Esposito, 2016). In this work, to assess differences and alterations of functional brain networks at both global and nodal scales caused by different lesion locations during cognitive processing, graph theoretical analysis was employed to compare the brain network reorganization across three groups. We focused on quantifying measures of network integration [e.g., characteristic path length (*L*), global efficiency (*E*_*glob*_)] and network segregation [e.g., clustering coefficient (*C*), local efficiency (*E*_*loc*_)]. Firstly, small-world networks with an optimal balance between global integration and local specialization were evaluated. The small-world metrics including *C*, *L*, and small-worldness (σ) were estimated in this study (Rubinov and Sporns, 2010).

First, weighted networks were constructed to preserve the relative strength information of connections, the weight of each connection was calculated by the wPLI method. For a given weighted network *W* with *N* nodes (*N* = 60 in this work), the weighted clustering coefficient quantifies the intensities of the subgraphs of node *i*, which is defined according to the proposal of Onnela et al. (2005):


(4) $$C_{i}=\frac{\sum_{k\neq i}\sum_{l\neq i,l\neq k}w_{ik}w_{il}w_{kl}}{\sum_{k\neq i}\sum_{l\neq i,l\neq k}w_{ik}w_{il}},$$

where *w* denotes the weight between two nodes. Then the mean clustering coefficient *C*, a global measure of functional segregation of the network, is computed as:


(5) $$\begin{array}{l} C=\frac{1}{N}\underset{i\in N}{\sum }C_{i}. \end{array}$$

The characteristic path length *L* is defined as the smallest sum of the shortest path length connecting all possible pairs of nodes:


(6) $$\begin{array}{l} L=\frac{1}{N\left(N-1\right)}\underset{i\in N}{\sum }\underset{i\neq j\in N}{\sum }L_{ij}, \end{array}$$

where *L*_*ij*_ denotes the shortest path length between node *i* and *j*. The path length of an edge in a weighted network is defined as the reciprocal of the edge weight. *L* quantifies the overall communication efficiency between any pair of nodes, is a global measure of functional integration of the network.

Small-worldness σ is a measure for quantitatively evaluating small-world properties (Rubinov and Sporns, 2010), which is defined as the ratio of the clustering coefficient to the characteristic path length after both metrics have been standardized through normalizing their values by those of equivalent random networks:


(7) $$\begin{array}{l} \sigma =\frac{C/C_{rand}}{L/L_{rand}}, \end{array}$$

where *C*_*rand*_ and *L*_*rand*_ indicate the mean weighted clustering coefficient and mean weighted characteristic path length derived from 100 matched random networks, which is constructed by randomly reshuffling the edge weights using the method according to Dimitrakopoulos et al. (2018).

Furthermore, to provide an explicit and direct physical meaning to the concept of small-worldness properties in terms of information flow, the efficiency of information transfer was calculated to measure the changes in network properties. Specifically, the *E*_*glob*_ and *E*_*loc*_ of brain networks were estimated (Latora and Marchiori, 2001, 2003). *E*_*glob*_ measures the network's capacity for parallel information transfer between nodes through multiple series of edges and is inversely related to *L*, which is computed as:


(8) $$\begin{array}{l} E_{\text{glob}}=\frac{1}{N\left(N-1\right)}\underset{i\neq j\in N}{\sum }\frac{1}{L_{ij}}, \end{array}$$

*E*_*loc*_ measures how well each subgraph exchanges information when the index node is eliminated, which is obtained as:


(9) $$\begin{array}{l} E_{\text{loc}}=\frac{1}{N}\underset{i\in N}{\sum }E_{glob}\left(i\right)=\frac{1}{N}\left(\frac{1}{N_{W_{i}}\left(N_{W_{i}}-1\right)}\underset{j,k\in W_{i}}{\sum }\frac{1}{L_{jk}}\right), \end{array}$$

where *N*_*W*_*i*__ is the number of nodes in the subgraph *W*_*i*_.

To determine the regional characteristics of functional brain networks, we additionally performed nodal analysis and assessed the regional engagement of different nodes within the brain network by evaluating the node strength (*Str*) of each node in the network, which is defined as the sum of the weights of edges connected to a certain node according to the following formula:


(10) $$\begin{array}{l} Str_{i}=\underset{j\in N}{\sum }w_{ij}, \end{array}$$

where *w*_*ij*_ indicates the weight of the edge between node *i* and *j*.

In a brain network, weak and non-significant edges may represent spurious connections (Rubinov and Sporns, 2010). Therefore, prior to the network analysis, a previously-validated sparsity threshold (defined as the ratio of the number of actual edges to the number of all possible edges in a fully connected network) was employed to remove those spurious connections and maintain the most intrinsic connections (Achard and Bullmore, 2007). Here, a wide sparsity range from 0.1 to 0.4 with a step size of 0.01 was adopted as mentioned in Kakkos et al. (2019), which obtained a series of weighted matrices. Subsequently, to reduce the dependence of statistical significance arising from the arbitrary choice of a specific threshold, the integrated graph theoretical metrics were estimated over the predefined range of sparsity. The integrated values were calculated based on the area under the curve for each metric (Gao et al., 2020). The calculation of those graph theoretic metrics was implemented based on the code of the Brain Connectivity Toolbox (Rubinov and Sporns, 2010).

### 2.6. Statistical analysis

Prior to the statistical analysis, the normality of all variables was assessed using the Shapiro-Wilk test, while homogeneity of variables was evaluated by Levene's test. Depending on the normality results, parametric or nonparametric statistical methods were applied in the following analysis. Behavioral performance, including reaction time (RT) and response accuracy (RA), was compared among three groups using the Kruskal-Wallis test in each task. The multiple comparison *post-hoc* analysis was performed to compare these two behavioral metrics between groups, with Bonferroni correction. In addition, a one-way ANOVA was performed to investigate differences in network metrics (i.e., *C*, *L*, σ, *E*_*glob*_, *E*_*loc*_, and *Str*) among the three groups. To identify specific differences, the Bonferroni *post-hoc* test was used. The Spearman's rank correlation test was applied to evaluate the relationship between behavioral RT and efficiency metrics for each group. In this study, statistical significance was set at *p* < 0.05, and all statistical analyses were performed by SPSS 26 software (IBM, New York).

## 3. Results

### 3.1. Behavioral performance

In Table 2, we showed the statistical analysis results of behavioral performance in each task. Given that the oddball task protocol was relatively simple, all participants performed well on average (mean RA > 0.96 for all groups in both tasks), indicating strong motivation. The statistical comparison showed the main group effect in both RT (*H* = 17.486, *p* < 0.001 in visual task, and *H* = 8.574, *p* = 0.015 in auditory task) and RA (*H* = 7.493, *p* = 0.024 in visual task, and *H* = 9.265, *p* = 0.010 in auditory task) in both tasks. Further *post-hoc* analysis showed that in the visual oddball task, RT was significantly shorter in the NC group than in the CS and SS groups (*p* = 0.019 and *p* < 0.001, respectively), while RA was significantly higher than that in the SS group (*p* = 0.046). Meanwhile, in the auditory oddball task, compared with the CS and SS groups, the NC group showed significantly shorter RT (*p* = 0.033 and *p* = 0.023, respectively) and higher RA (*p* = 0.048 and *p* = 0.017, respectively). Significantly worse behavioral performance in both patient groups implied cognitive impairment in visual and auditory information processing. Moreover, on the basis of *post-hoc* analysis, there were no significant differences in behavioral performance between the two patient groups in both tasks.

**Table 2:** Statistical comparison of behavioral results in each oddball task.

		**Kruskal-Wallis test**				
		**NC**	**CS**	**SS**	***H***	***p-value***
		**Mean** ±**SD**	**Mean** ±**SD**	**Mean** ±**SD**				
Visual task	RT	323.260 ± 45.110^†^, ^‡^	366.390 ± 82.805	374.777 ± 77.454	17.486	**< 0.001**
	RA	0.990 ± 0.013^‡^	0.976 ± 0.037	0.978 ± 0.032	7.493	**0.024**
Auditory task	RT	253.728 ± 60.123^†^, ^‡^	309.547 ± 123.077	311.229 ± 132.505	8.574	**0.015**
	RA	0.992 ± 0.011^†^, ^‡^	0.979 ± 0.031	0.963 ± 0.063	9.265	**0.010**

RT means reaction time, RA means response accuracy.

† Means significant differences between the NC and CS groups after multiple comparison test (*p*-value corrected by Bonferroni).

‡ Means significant differences between the NC and SS groups after multiple comparison test (*p*-value corrected by Bonferroni).

Significant differences (*p* < 0.05) are indicated by the bold font.

### 3.2. Analysis of global network properties

**Figure 2:**
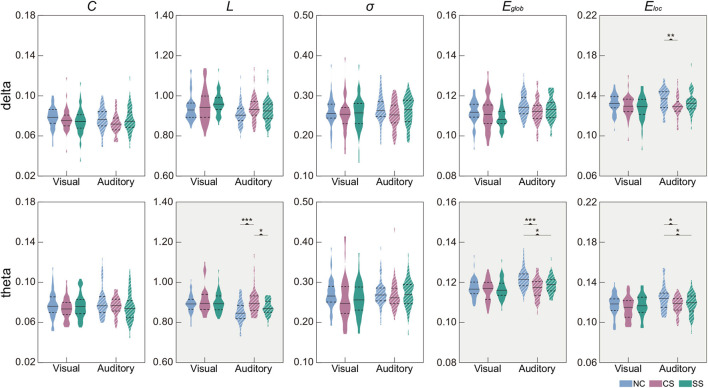
Distribution of global metrics in each group in the delta and theta frequency bands. Metrics with the significant main effect of group were indicated by a gray background. The first and third quartiles are indicated by dashed black lines, and the median is indicated by a solid black line. *Post-hoc* tests were calculated with Bonferroni correction for multiple comparisons. Asterisks indicate those pairs with statistically significant differences (^*^p < 0.05;^**^p < 0.01;^***^p < 0.001).

In Table 3, we showed the statistical results of global metrics in four frequency bands. In the visual task, no significant global metric differences were found across the three groups in all frequency bands. In the auditory task, however, a significant main effect of group was revealed in the delta and theta bands. Specifically, a significant main group effect was shown in *E*_*loc*_ (*F*_2,135_ = 4.639, *p* = 0.011) in the delta band, while *L* (*F*_2,135_ = 10.869, *p* <0.001), *E*_*glob*_ (*F*_2,135_ = 10.829, *p* <0.001), and *E*_*loc*_ (*F*_2,135_ = 5.342, *p* = 0.006) presented significant difference among the groups in the theta band. The results of further *post-hoc* analysis for those two frequency bands were illustrated in Figure 2. In the theta band, the *L* of the NC group was significantly shorter than that of the CS group (*p* <0.001) while the value of the CS group was significantly longer than that of the SS group (*p* = 0.024). Regarding the efficiency metrics, in the delta band, the *E*_*loc*_ was significantly reduced (*p* = 0.008) in the CS group than that derived in the NC group, while in the theta band, significantly decreased *E*_*loc*_ was not only found in the CS group (*p* = 0.011) but also shown in the SS group (*p* = 0.031). Although the *post-hoc* analysis showed no statistically significant difference between the SS group and NC group in the delta band, a decreasing trend of *E*_*loc*_ was exhibited in the SS group. In addition, the *E*_*glob*_ of the NC group was significantly higher than that of the CS group (*p* <0.001) and SS group (*p* = 0.010) in the theta band. A similar decreasing trend of *E*_*glob*_ was also found in the delta band of both patient groups though there was no significant difference.

**Table 3:** One-way ANOVA results of global metrics.

**Newtork metrics**		**delta**		**theta**		**alpha**		**beta**	
		*F* **-value**	*p* **-value**	*F* **-value**	*p* **-value**	*F* **-value**	*p* **-value**	*F* **-value**	*p* **-value**
Visual task	*C*	1.507	0.225	1.027	0.361	0.609	0.545	1.708	0.185
	*L*	1.283	0.281	1.484	0.230	0.339	0.713	0.790	0.456
	σ	0.182	0.834	1.088	0.340	0.978	0.379	1.048	0.353
	*E*_*glob*_	2.440	0.091	0.409	0.665	1.197	0.305	0.420	0.658
	*E*_*loc*_	1.790	0.171	1.378	0.256	1.141	0.323	0.910	0.405
Auditory task	*C*	2.099	0.127	1.780	0.173	1.152	0.319	1.583	0.209
	*L*	2.927	0.057	10.869	**< 0.001**	0.740	0.479	2.470	0.088
	σ	1.932	0.149	0.176	0.838	0.096	0.909	0.652	0.523
	*E*_*glob*_	2.868	0.060	10.829	**< 0.001**	0.462	0.631	0.902	0.408
	*E*_*loc*_	4.639	**0.011**	5.342	**0.006**	1.276	0.283	2.264	0.108

Significant differences (*p* < 0.05) are indicated by the bold font.

### 3.3. Analysis of nodal network properties

**Figure 3:**
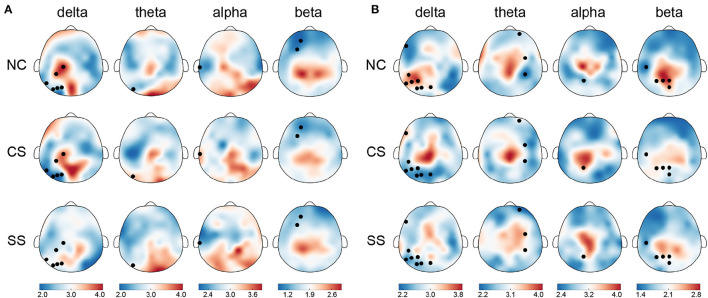
The topographical maps of node strength for each node in each group in four frequency bands in **(A)** the visual task and **(B)** the auditory task. Node with significantly different node strength (*p* < 0.05) in one-way ANOVA is highlighted in black. The color bar indicates mean node strength values.

In terms of nodal metrics, we found distinct spatial-frequency patterns across three groups in both tasks, as depicted in Figure 3. In the visual task, node strength differences mostly appeared in the delta frequency band (six out of 10), mainly distributed in the parietal and occipital lobes of the left hemisphere. Meanwhile, in the remaining three frequency bands, the distribution with significant differences in node strength (one in theta, one in alpha, and two in beta frequency bands) was also concentrated in the left hemisphere. In the auditory task, similar to the distribution in the visual task, the largest number of nodes with significant differences in node strength were exhibited in the delta band (eight out of 17), mainly located in the temporal, parietal, and, occipital lobes of the left hemisphere. However, the distribution in the theta band (three out of 17) was located in the right hemisphere. In the alpha and beta bands, statistically significant nodes of node strength (one in alpha, and five in beta frequency bands) appeared in the left hemisphere which was predominantly from the parietal region.

In addition to the differential distribution of spatial frequency patterns within each task, we also found common distribution patterns in the two patient groups. Specifically, in the visual task, in each group, the nodes with stronger node strength were mainly located in the parietal and occipital lobes in the delta, theta, and alpha frequency bands, while in the beta band, they were predominantly located in the parietal lobe. In the auditory task, there was also a common distribution pattern between the two groups of patients. In the theta, alpha, and beta frequency bands, the distribution of nodes with stronger node strength mainly came from central and parietal regions.

### 3.4. Correlation between global metrics and behavioral performance

**Figure 4:**
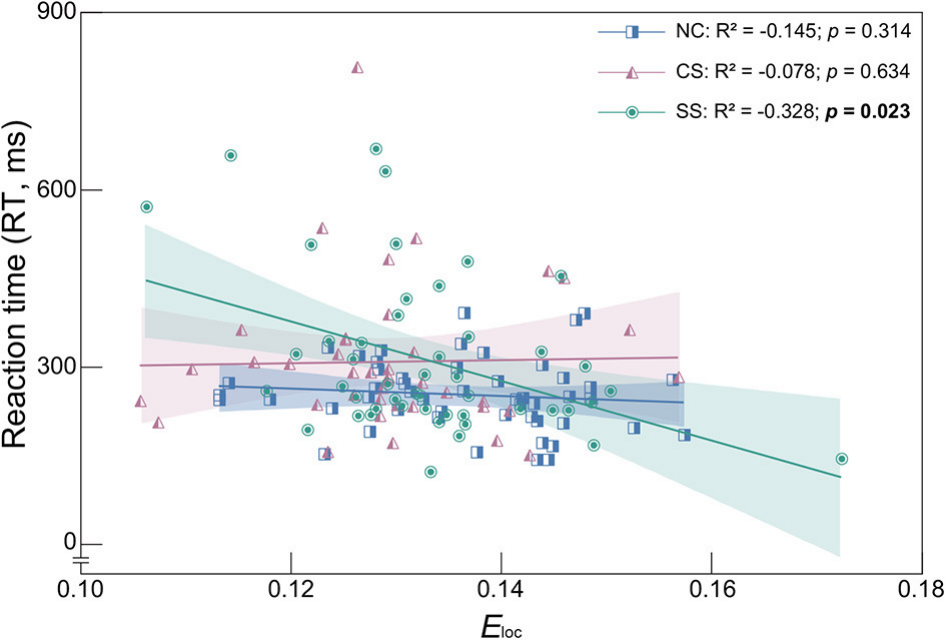
Spearman's correlation coefficient and linear regression analysis between *E*_*loc*_ and RT in the delta frequency band of each group. *R*^2^ denotes the Spearman correlation coefficient. Colored area represents the 95% confidence interval. Correlation with statistically significant is indicated by the bold text.

To determine the relationship between the behavioral measures and global network measures, metrics with statistically significant differences were employed in the following correlation analysis (i.e., *E*_*loc*_ in the delta band, *L*, *E*_*glob*_ and *E*_*loc*_ in the theta band, and RT in the auditory oddball task). The results with significant correlation were shown in Figure 4. In the SS group, the *E*_*loc*_ in the delta band showed a significant negative correlation with RT (*R*^2^ = −0.328, *p* = 0.023). Therefore, for patients with subcortical lesions, a higher value of *E*_*loc*_ in the delta band corresponded to a faster response speed in the auditory oddball task. However, in the CS group, there was no significant correlation between these two metrics, nor in the NC group.

## 4. Discussion

To the best of our knowledge, this is the first study focusing on the impact of different lesion locations on functional brain network characteristics during cognitive processes in patients with mild stroke. To that end, a widely-used cognitive paradigm involving two modalities of information processing (i.e., visual and auditory oddball tasks) was employed. Subsequently, the alterations in topological network properties were quantitatively estimated via graph theoretical analysis. The significant findings are as follows:

• Significantly worse behavioral performance was found in patient groups, suggesting that the impact of mild stroke on information processing is lesion location-independent.
• At the global level, the patient groups showed brain network reorganization during the auditory task, implying impaired efficiency of information transfer during cognition.
• At the nodal level, complex distributions of significantly different node strength were found, whereas a similar distribution pattern was shown in patient groups. The results suggest that different lesion locations induce complex regional involvement and overlapping cognitive processes.

These findings were mostly consistent with our hypothesis that patients with mild stroke often have cognitive deficits that manifested in behavioral performance and brain network characteristics, and lesion location would lead to differential topological changes. The specific discussion is as follows.

### 4.1. Similar patterns of worse behavioral performance in both patient groups

The oddball paradigm has been frequently employed to investigate the mechanism of attention (Akimoto et al., 2013), meanwhile, attention deficits have been shown to persist over time in stroke patients (Hyndman et al., 2008). Thus, as expected, both patient groups performed significantly worse on the two oddball tasks, exhibiting similar behavioral patterns, which was in line with the previous studies (Saygın et al., 2003; Shan et al., 2018). In a study of patients with right hemisphere stroke, spatial attention processing was investigated using a visual oddball task. Behavioral performance was significantly worse regardless of whether the target stimulus was presented on the left or right, suggesting information processing deficits in patients (Shan et al., 2018). Our findings in the visual task further support the notion that information processing function may be impaired in stroke patients given the patients in our research were all with mild symptoms. In addition, similar deficits in auditory information processing were also found in stroke patients. By instructing participants to listen to the sound and press the response button as quickly as possible, Saygın et al. (2003) assessed the relationship between verbal and nonverbal auditory processing in left hemisphere-damaged stroke patients with varying severity of aphasic. The patient group tended to perform worse on both types of auditory stimuli, suggesting that stroke patients were impaired in the processing of both meaningful verbal and nonverbal auditory information. The current work extended previous research to mild strokes and further strengthened previous findings on the dysfunction of nonverbal auditory information processing. Of note, although we divided patients into two subgroups based on lesion location, the patients recruited in this work were all with mild stroke. Our findings extended previous studies which mainly focused on patients with moderate-to-severe stroke to mild stroke, meanwhile, provided new behavioral evidence that visual and auditory information processing impairments were common cognitive dysfunctions in patients with mild stroke independent of varying lesion locations.

### 4.2. Differential patterns of global network characteristics between groups

In general, brains have high global and local efficiency, representing functional integration and segregation (Latora and Marchiori, 2001; Bullmore and Sporns, 2009). Decreased global and local efficiency have been found in stroke patients in the auditory oddball task, indicating functional reorganization of peripheral and remote brain regions and disruption of cluster connections among topologically nearby neighbors (Fallani et al., 2013; Dacosta-Aguayo et al., 2015). Although alterations in functional integration and segregation have been repeatedly reported in stroke patients (De Vico Fallani et al., 2009; Wang et al., 2010), significant alterations were only found in the auditory task. Differing from visual deficits in patients with stroke, auditory processing deficits are overshadowed by more prominent symptoms involving other neurological systems (Häusler and Levine, 2000). Previous studies have repeatedly demonstrated that stroke patients often report intact hearing, yet the majority of these patients fail the hearing screening (Edwards et al., 2006b; Bamiou et al., 2012). Given that the patients recruited in this work were those who self-reported without obvious hearing deficiency, we inferred that the significant differences in information transfer efficiency might be due to inconspicuous deficits in auditory function in patients with mild stroke.

The prominent role of delta- and theta-band connectivity between distributed cortical regions during attention, stimulus detection, and response selection processing may explain the significant global and local efficiency reduction in patient groups (Cavanagh and Frank, 2014; Gulbinaite et al., 2014). On the one hand, the oddball task processing involves signal detection and decision-making functions that are thought to be associated with the delta band (Başar-Eroglu et al., 1992; Pornpattananangkul et al., 2019). On the other hand, reduced local efficiency has been reported to be related to increased cognitive effort (Kitzbichler et al., 2011). These may indicate that stroke patients expend more effort to detect auditory target stimuli and make decisions during the task. Of note, there are strong links between the theta band and cognitive processes, including sustained attention, and executive function (Mitchell et al., 2008; Kawasaki et al., 2014). Youssef et al. (2021) assessed functional brain network alterations in mild cognitive impairment (MCI) patients and found the alterations in global and local efficiency were frequency-specific in the theta band, which was classically related to attention processes. In addition, consistent with previous studies in patients with MCI, significant reductions in efficiency measures were associated with network disintegration in stroke patients (Wang et al., 2018). We, therefore, speculate that frequency-specific network disintegration in patient groups may be due to impaired auditory attentional function. Notably, in tasks requiring complex manipulations, theta coupling between prefrontal and parietal cortices is increased, suggesting recruitment of executive control functions (Sauseng et al., 2005). Moreover, theta coherence between the frontal cortex and the temporal-parietal cortex was found to increase with memory load (Payne and Kounios, 2009). Theta synchronization plays a crucial role in large-scale communication and information transfer between distant brain regions (Fell and Axmacher, 2011). Thus, the significantly reduced efficiency of information transfer in patients with mild stroke suggests a disturbance in large-scale communication, which may further support the idea that focal lesions would affect distant brain regions albeit with mild stroke symptoms. Additionally, consistent with previous studies (Caliandro et al., 2017; Fanciullacci et al., 2021), functional integration of the network as reflected by *L* in the theta band was reduced in both patient groups, suggesting that patients with mild stroke have dysfunction in integrating information and transmitting information over long distances during auditory information processing. Overall, functional disintegration was found in both patient groups during the auditory task, which indicated that disruption of functional brain networks during cognitive processing may be affected by mild strokes independent of lesion location. Although visual dysfunction is a common sequela in stroke patients, it is often accompanied by obvious symptoms (Hanna et al., 2017). Note that all patients recruited in this study had normal vision and no apparent visual deficits, which may help explain no significant differences in the visual task. Furthermore, network metrics derived from an abstract global perspective may obscure subtle changes given the worse behavioral performance was found in the visual task, which may be another possible explanation. In conclusion, distinct patterns of global topological alterations across groups were revealed during the auditory task, indicating different information processing strategies elicited by mild stroke.

### 4.3. Complex node network properties in patient groups

Essentially, the predominant distribution of regional engagement in the delta band could indicate changes in cognitive demands for signal detection and decision making (Dolce and Waldeier, 1974; Başar-Eroglu et al., 1992). Moreover, there is growing evidence that stroke patients often have deficits in decision-making (Poulin et al., 2013; Scheffer et al., 2016). In the oddball tasks, at the behavioral level, task performance is associated with signal detection and decision-making. Therefore, both patient groups performed worse on the task, reflecting the increased cognitive demand imposed by signal detection and decision-making. Numerous studies have shown that attention functions can be selectively damaged as a function of one side of the lesion (Fan et al., 2005; Vanderhasselt et al., 2009). In addition, a considerable amount of lesion location and neuroimaging research has shown that sustained attention processes are associated with right hemisphere cortical and subcortical networks (Sturm and Willmes, 2001), whereas left hemisphere mechanisms are involved in some more complex attention functions, including selective, executive and temporal attention (Fan et al., 2005; Vanderhasselt et al., 2009). However, several studies comparing stroke patients with right or left hemisphere lesions failed to find significant between-group differences in simple RT and sustained attention (Bub et al., 1990; Audet et al., 2000). In our study, differences in the degree of regional engagement were prominently localized in the left hemisphere, as oddball tasks are one of the sustained attention paradigms, this lateralized distribution implied attention deficits in both visual and auditory information processing in stroke patients.

Interestingly, a common distribution pattern of node strength was found in the two patient groups in each task. Specifically, nodes with higher strength appeared predominantly in the parietal lobe in both tasks. Indeed, the parietal region plays an important role in visual attention (Bueichekú et al., 2015; Wang et al., 2020a). Meanwhile, accumulating evidence suggests that the parietal lobe is not only involved in visual attention but also in auditory attention (Shinn-Cunningham et al., 2017; Jacquemot and Bachoud-Lévi, 2021). In the oddball paradigms, the parietal region is often activated by infrequent target stimuli that elicit brief stimulus-driven shifts of attention (Igelström and Graziano, 2017). Consistent with recent studies (Lewald et al., 2018; Jurewicz et al., 2020), activation of the parietal lobe was found during the oddball task with both visual and auditory stimuli, as evidenced by the distribution of nodes with the highest strength. As a similar distribution was found in both patient groups, convergent changes in regional engagement may indicate that the task-induced shift of attention was independent of lesion locations.

### 4.4. Relationship between network efficiency and behavioral performance

Reaction time is considered a good indicator of the speed and efficiency of mental processes (Draheim et al., 2019), whereas local efficiency measures the functional segregation, the capacity of locally processed information (Latora and Marchiori, 2001). Even though some neurons are damaged, brain networks with high local efficiency are robust in local information processing (Huang et al., 2014). Therefore, we speculate that the higher the local efficiency, the faster the reaction time, indicating that the stronger the local information transmission ability, the faster the cognitive process. However, opposite findings were shown in a prior study that investigate the relationship between non-task-related resting-state functional brain network topology and reaction time in a Go/Nogo task in healthy individuals. The results indicated that the increased efficiency of local information integration would result in slower sensorimotor processing, as reflected by a negative correlation between reaction time and clustering coefficient (Zhou et al., 2012). Although these findings provide evidence of the association between the functional brain network and cognitive function, it should be noticed that it was focused on healthy college students, and the network metrics were estimated utilizing resting-state functional connectivity. Recently, using within-group comparisons, Shang et al. (2021) examined the effect of handgrip training on cognition in patients with acute mild stroke based on diffusion tensor images, and they found that improvements in cognitive function were accompanied by increased local efficiency. The differences in the study subjects may explain the contrary results of previous studies on the relationship between local efficiency and cognitive function. In the present work, our findings were consistent with prior research on acute mild stroke, suggesting that the value of local efficiency may be an effective variable in predicting performance during cognitive tasks in patients with mild subcortical stroke.

### 4.5. Future consideration

In this work, some issues should be considered when interpreting our findings. First, given the inherently heterogeneous characteristics of stroke patients, different lesion hemispheres and stroke time may distort alterations in brain network properties in patient groups. Previous research has shown that sustained and intrinsic attentional functions are associated with the right hemisphere (Sturm and Willmes, 2001), whereas phasic alertness is associated with the left hemisphere (Fan et al., 2005). However, there were some contrary findings as the researchers compared reaction time, sustained attention, and phasic alertness in patients with right-hemisphere lesions and left-hemisphere lesions and found no significant differences between the two groups (Bub et al., 1990; Audet et al., 2000). Moreover, the impact of stroke time on attentional function remains controversial (Hyndman et al., 2008; Barker-Collo et al., 2010). Here, additional statistical analyses were performed to examine the effects of lesion hemispheres and stroke time, and the main outcome was found to be intact. Nevertheless, further studies with larger sample sizes should be considered to replicate our findings. Second, to maintain the task cooperation of older participants as well as the ability to reveal intrinsic cognitive function in patients with mild stroke, our study employed two widely-used simple cognitive tasks. It should be noted that a large number of studies have indicated that cognitive impairment is a common consequence of stroke and may hinder the rehabilitation and functional recovery of stroke patients (Tatemichi et al., 1994; Patel et al., 2002). The most frequently impaired cognitive processes include attention, information processing, memory, and executive function (Tatemichi et al., 1994; Ballard et al., 2003). Neuropsychological examination of cognitive functions in patients with cortical and subcortical stroke revealed that patients with subcortical lesions performed worse cognitive profiles compared to patients with cortical lesions, including verbal memory, executive functions, and psychomotor speed (Turunen et al., 2013). The present work further explored the cognitive function of patients with different lesion locations through a neurophysiological approach and found no significant difference between cortical and subcortical patients, which may be due to the fact that the patients in this study were all mild strokes. Furthermore, the relatively simple task paradigm may be another explanation, which could be limited in detecting deeper and more complex cognitive functions (Sivák et al., 2008; Paitel et al., 2021). Thus, further studies on mild stroke patients with different lesion locations could employ slightly more complex cognitive paradigms such as Go/No-Go, and Stroop paradigms.

## 5. Conclusion

In this study, we investigated differences and commonalities in functional brain networks during cognitive processes between mild cortical and mild subcortical strokes based on graph theoretical analysis. The results showed that patients with mild cortical and subcortical strokes had deficits in both visual and auditory information processing, as manifested by significantly worse behavioral performance. In addition, the disruption of global and local information transmission and the similarities and differences in node distribution patterns reflect the complexity of information processing caused by different lesion locations, as well as the commonality of cognitive processes independent of lesion location. In sum, this work extends previous studies on moderate-to-severe stroke to mild stroke and reveals divergent and convergent patterns of network reorganization during cognition in patients with cortical and subcortical stroke. Furthermore, these findings may contribute to understanding the altered neural mechanisms in patients with different lesion locations and provide heuristic information to facilitate further investigations in mild stroke rehabilitation.

## Data availability statement

The data analyzed in this study is subject to the following licenses/restrictions: Data used in the work was available upon reasonable request to the corresponding author. Requests to access these datasets should be directed to YuS, yusun@zju.edu.cn.

## Ethics statement

The studies involving human participants were reviewed and approved by the Institutional Review Board of the Sir Run Run Shaw Hospital, Zhejiang University School of Medicine. The patients/participants provided their written informed consent to participate in this study.

## Author contributions

XQ and YuS: conceptualization, resources, and funding acquisition. MX, LQ, SW, HC, YiS, NT, XQ, and YuS: methodology. MX: formal analysis. MX and YuS: writing—original draft preparation. YuS: supervision. All authors have read and agreed to the published version of the manuscript.
